# Microwave Induced Plasmas as Sources for Atomic Spectrometry

**DOI:** 10.6028/jres.093.114

**Published:** 1988-06-01

**Authors:** Joseph A. Caruso

**Affiliations:** Department of Chemistry, University of Cincinnati, Cincinnati, OH 45221

## 1. Introduction

Microwave induced plasmas (MIPs) have been studied For their potential applicability as analytical sources for over 20 years. In 1965 the MIP was described as an elemental emission detector for gas chromatography (GC) by McCormack and coworkers [1]. Because of its initial low power operation of less than 100 Watts, this plasma, with He primarily as the plasma gas, has been most successfully applied for introduction of gaseous samples. Hence, it is understandable that GC sample introduction [2], hydride generation [3], electrothermal vaporization [4] and other means of gaseous sample introduction have been successfully applied with the MIP. Until recently, solution aerosol introduction was not a particularly viable means of introducing sample to the MIP. However, with the use of the more robust MIPs of up to 500 Watts of generator power [5,6], good analytical figures of merit have been obtained with aerosol introduction of samples.

This presentation discusses the use of Ar and He microwave induced plasmas as spectrochemical sources for gaseous sample introduction and with solution nebulization methods of sample introduction. The first topic focuses on the application of the GC technique; the accuracy available through determination of elemental ratios; and the resulting empirical formulae for a series of control compounds for which empirical formulae have been established. Low level detection at the ng to sub-ng levels is available, however, the comparisons with standard materials to establish accuracy are not prevalent in the literature, although hydride generation has been utilized with NBS Orchard Leaves [3]. The discussion involving solution introduction through nebulized aerosols will consider both optical emission detection and mass spectrometric detection [7,8]. With both of these schemes, a variety of standard reference materials (SRMs) or alternate method comparisons can be made.

## 2. Gaseous Sample Introduction into MIPs

**Figure 1:**
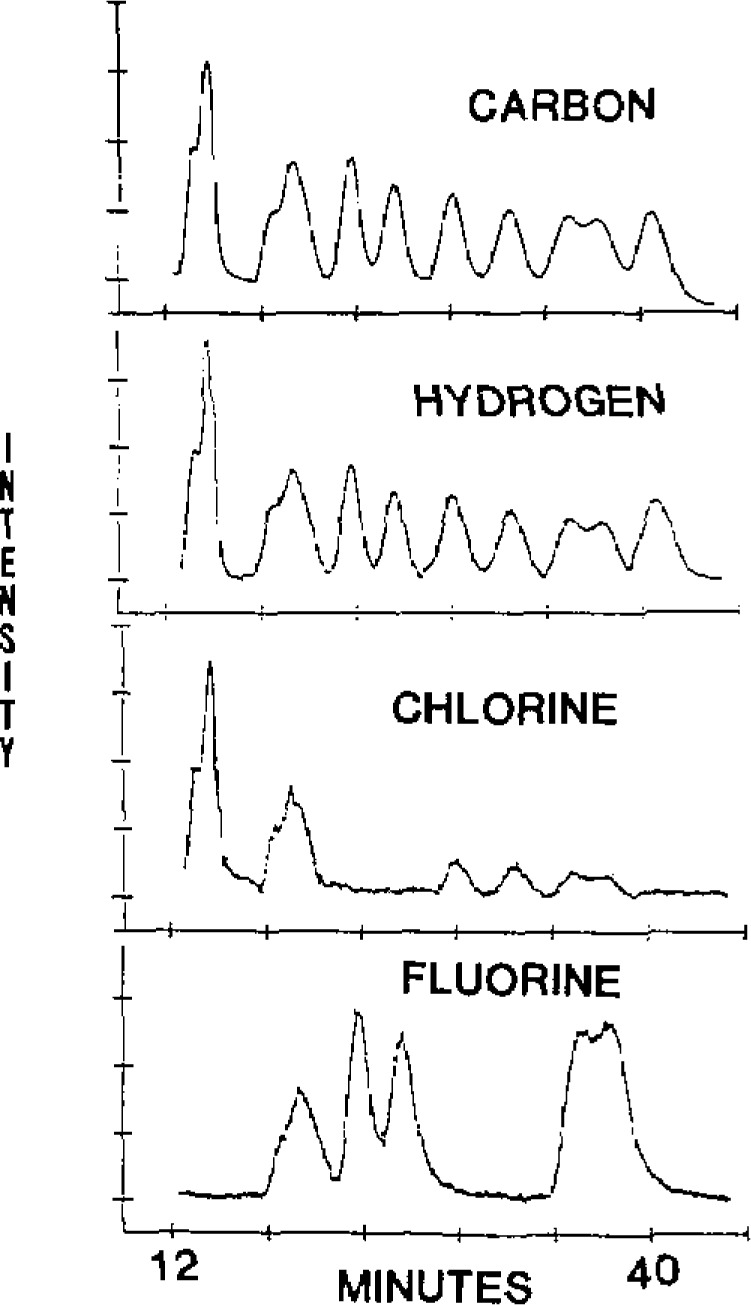
Background-corrected chromatograms of the pyrethroids taken with the LFT for carbon, hydrogen, chlorine, and fluorine channels Permethrin, Cyfluthrin, Flucythrinate (2 peaks), Fenvalerate (2 peaks), Fluvalinate (double peak), and Deltamethrin.

Element specific detection for GC has the advantages of low level detection, empirical formula determination and potential to overcome problems from lack of chromatographic resolution. The latter is because each element’s optical emission or elemental mass may be monitored simultaneously or in rapid sequence. With MIP detection of organic compounds, C or H can serve as universal detectors whereas halogens, phosphorous, sulfur, oxygen and other elements can provide element specific chromatograms. This potential for overcoming problems associated with peak overlap is illustrated in figure 1 [9]. Multielement simultaneous detection is done through use of a polychromator [9]. In these instances, each chromatographic peak is monitored for the various elements contained in the eluting compound. If only single element detection is required, then the experiment can be simplified through the use of a modest monochromator.

One of the areas explored in recent years involves the use of a laminar flow plasma torch (LFT) to replace the open tube quartz torch which has been the most popular plasma containment device. Plasma stability is critical to performing the GC-MIP experiment. By producing a laminar gas flow, the plasma remains centered and therefore optical coupling is much more effective since plasma wander is minimized [9]. With this torch, optical emission detection levels at the sub-ng levels are possible. Low gas flows on the order of 60 mL/min are necessary. Plasma centering also minimizes torch degradation—an essential requirement for the laboratory analyst. Empirical formulae can be determined if the appropriate elemental ratio is determined. Partial empirical formulae for a series of chlorodioxins determined by GC-MIP with the LFT are given in table 1 [9]. A more comprehensive table is given in [2] which includes many contributions from a large number of workers. These rather extensive data suggest suitable accuracy is possible for empirical formula determination with GC-MIP even in a widely varying chemical (structural) environment.

Certain elements form volatile hydrides when treated with sodium borohydride. Robbins and Caruso investigated the use of these for gaseous sample introduction of the As and Sb volatile hydrides as applied to NBS SRM 1571 Orchard Leaves, These data are presented in table 2 [3]. These data show that it is possible through the gaseous (hydride) sample introduction to the MIP to achieve accuracies within acceptable ranges for SRM 1571.

## 3. Solution Nebulization for Sample Introduction to the MIP

Studies by Haas [5], and Brown et al. [6] have shown that sufficient plasma density is produced when operating at 500 Watts generator power to allow solution introduction of samples into the Ar MIP for optical emission. Other workers have illustrated that with desolvation even a 180 Watt plasma can yield useful analytical data with mass spectrometric detection [8]. With low power MIPs, solution nebulization gave detection levels one to two orders of magnitude poorer than those available from the ICP. In addition, the tolerance to matrix interferences was poor. At the low powers, the plasma was simply not robust enough to accommodate desolvation as well as excitation, particularly in the presence of solvent vapor which acts to detune the plasma. At the higher power levels, however, enough energy is efficiently coupled to the flowing gas stream to achieve much better analytical performance with the plasma.

With a direct solution nebulization, the moderate power MIP was studied to ascertain its suitability for metal ion determinations in solution. Discharge power, plasma viewing mode, cavity depth, nebulizer type and discharge containment mode were investigated [5]. Cu, Al, Pb, Cr, Mn, Fe, Hg, Cd, Zn, Mg, and Ni were investigated in 2% HNO_3_ solution as well as a synthetic ocean water (SW) with nearly 3% dissolved solids. For the acid solutions, the detection levels paralleled those of the ICP at low ppb levels, while in the SW, the ion lines provided poorer detection and the atom lines still afforded excellent detection levels. Additional studies with NBS SRM 1577 Bovine Liver showed good agreement with the certified values. Further studies provided a comparison with two ICPs. Again, the comparison was very good in these experiments. One of the ICP studies was done at the local FDA laboratories and the other with the Department of Chemistry ICP. Table 3 illustrates these comparisons.

With solution introduction into the MIP possible, Douglas and co-workers [8] did the first MIP-MS studies with a 180 Watt Ar plasma. Their results were excellent, however, no further MIP-MS studies were completed until recently [7], Douglas’ work is particularly pertinent to this discussion since this study utilized comparisons with a number of SRMs. NBS SRM 362 Steel, AISI, was analyzed by both a simple calibration plot and by the method of standard additions. For the elements studied, the standard addition experiments provided the best comparisons with certified values. They also studied trace elements in KBS SRM 1643a Trace Elements in Water. Simple calibration in most cases was unsuitable while standard additions or matrix matching provided a much better comparison with certified values. Of the latter two, it would be difficult to say which of them is better. These authors also studied trace elements in SRM 1571 Orchard Leaves by MIP-MS and showed that good comparisons were possible if the standard additions technique was used.

In summary, microwave induced plasmas are capable optical emission and plasma mass spectrometrie sources. If properly applied, they can yield excellent analytical data giving both accurate and precise results at the trace elemental level.

## Figures and Tables

**Table 1:** Partial dioxin empirical formulas obtained with the LFT

Compound	Partial empirical formulas		Error	
	Actual	GC-MIP	Atom^a^	amu^a^
2,7-CDD	C_6_H_3_Cl	C_6_H_3_Cl	0%	0%
1,2,4-CDD	C_12_H_5_Cl_3_	C_12_H_5_Cl_5_	0%	0%
1,2,3,4-CDD	C_3_HCl	C_3_HCl	0%	0%
1,2,4,6,7,9-CDD	C_6_HCl_3_	C_6_H_2_Cl_3_	33%	1%
1,2,3,4,6,7,9-CDD	C_12_HCl_7_	C_11_HCl_7_	3%	3%
1,2,3,4,6,7,8,9-CDD	C_3_Cl_2_	C_3_Cl_2_	0%	0%

a See text for explanation, Appl. Spectrosc. 39, 948 (1985), ref. 9.

**Table 2:** Comparison of values obtained for NBS Orchard Leaves, Lot 1571^c^

	Value given μg/g	Value detmd. sequentially μg/g	Value detmd. simultaneous μg/g
As	10.0±2^a^	9.0±4^b^	8.9± 1.2^b^
Sb	2.9 ±0.3^a^	2.3±0.3^b^	2.9±0.3^b^

a Two standard deviations “of entire range of observed results.”

b Average deviation of four determinations.

c From reference [3].

**Table 3:** Analysis of NBS SRM 1577 Bovine Liver by MIP and ICP^d^

Element	Certified value, ppm	MIP, ppm	% RSD	% difference with certified	ICP,^a^ ppm	% RSD	% difference with certified	ICF,^b^ ppm	RSD	% difference with certified
Cu	1.27± 10%	1.37	1.4	8.08	1.34	2.3	5.7	1.3	ND^c^	4.2
Fe	2.29+20%	2.21	1.9	3.67	2.36	4.0	2.9	2.6	ND	10.6

a Leeman Labs Plasma Spec ICP 2.5, university laboratories.

b Jarrell Ash Atom Comp 1140, US FDA.

c Not determined.

d From reference 6.
